# Percutaneous transesophageal gastro-tubing for the management of anastomotic leakage after upper GI surgery: a report of two clinical cases

**DOI:** 10.1186/s40792-020-00965-z

**Published:** 2020-08-24

**Authors:** Yutaka Tamamori, Katsunobu Sakurai, Naoshi Kubo, Ken Yonemitsu, Yasuhiro Fukui, Junya Nishimura, Kiyoshi Maeda, Yukio Nishiguchi

**Affiliations:** 1 Department of Gastroenterological Surgery, Osaka City General Hospital, 2-13-22, Miyakojima-hondori, Miyakojima-ku, Osaka, 534-0021 Japan; 2 Department of Surgery, Osaka City Juso Hospital, 2-12-27, Nonaka-kita, Yodogawa-ku, Osaka, 532-0034 Japan

**Keywords:** Percutaneous transesophageal gastro-tubing, Anastomotic leakage, Upper GI surgery, Transnasal drainage, Double-lumen feeding tube

## Abstract

**Background:**

Anastomotic leakage is a serious, sometimes critical complication of upper gastrointestinal (GI) surgery. The cavity and target drainage tubes are difficult to reach; therefore, a nasogastric tube (NGT) and fasting are required for an extended period. We successfully treated and managed two patients with anastomotic leakage using percutaneous transesophageal gastro-tubing (PTEG).

**Case presentation:**

In case 1, a 79-year-old man with gastric cancer underwent total gastrectomy; 1 week later, he underwent emergent open laparotomy due to panperitonitis attributed to anastomotic leakage-related jejunojejunostomy. We resected the portion between esophagojejunostomy and jejunojejunostomy and reconstructed it using the Roux-en-Y technique. On postoperative day (POD) 9, anastomotic leakage was diagnosed at the esophagojejunostomy site and jejunotomy staple line. After using a circular stapler for jejunojejunostomy, a stapled jejunal closure was added. We inserted an NGT and performed aspiration for bowel decompression. As he did not improve within 2 weeks, we decided to perform PTEG to free him of the NGT. We kept performing intermittent aspiration; leakage stopped shortly after, due to effective inner drainage. The PTEG catheter was removed after oral intake was restarted. In case 2, an 81-year-old man with esophagogastric junction cancer underwent resection of the distal esophagus and proximal stomach. After shaping the remnant stomach, esophagogastrostomy was performed under the right thoracotomy. On POD 11, anastomotic leakage was identified, along with a mediastinal abscess. We inserted an NGT into the abscess cavity through the anastomotic leakage site. On POD 25, we performed PTEG and inserted a drainage tube, instead of an NGT. Although the abscess cavity disappeared, anastomotic leakage persisted as a fistula. We exchanged the PTEG with a double elementary diet (W-ED) tube with jejunal extension, with the side hole located near the anastomosis. The anastomotic fistula disappeared after treatment. Dysphagia persisted due to disuse atrophy of swallowing musculature; PTEG was useful for enteral feeding, even after the leakage occurred.

**Conclusion:**

Patients are sometimes forced to endure pain for a long time for transnasal inner drainage. Using PTEG, patients will be free of sinus pain and discomfort; PTEG should be helpful for patients withstanding NGT.

## Background

Percutaneous transesophageal gastro-tubing (PTEG) is a non-surgical technique developed to access the stomach via an esophagostomy [1]. It is established as a palliative care procedure for patients with malignant bowel obstruction who could not undergo percutaneous endoscopic gastrostomy (PEG) [2–8]. The tubing procedure applied for enteral feeding for patients with contraindications to gastrostomy is widespread in Japan [9–13].

When the anastomotic leakage occurred after upper gastrointestinal (GI) surgery, effective drainage is required. However, it is often difficult to reach a catheter into the abscess cavity. Sometimes, colorectal surgeons perform transanal drainage to decompress the rectum [14–22]. It can be one of the options when we want to avoid repeated surgical treatments for some reason; it may also be useful for upper GI surgery when performed for esophagogastric or esophagojejunal anastomotic leakage. It is certainly possible to perform proper drainage using a nasogastric tube (NGT); conversely, we force the patients to endure pain for a long time.

Herein, we report two cases using PTEG as an alternative of NGT for the management of anastomotic leakage after upper GI surgery, and we propose the procedure as one of the usual non-surgical clinical options.

## Case presentation

### Procedure

PTEG is performed for patients with anastomotic leakage when there are concerns about the NGT remaining for over 2 weeks, even if the purpose of the tubes is for feeding, bowel decompression, or abscess drainage. Informed consent and anticoagulant discontinuation confirmation were obtained before the procedure. We used the PTEG kit supplied by Sumitomo Bakelite Co. Ltd., Tokyo, Japan. We followed the procedure of the original method by Oishi et al. [1]. The kit comprises a rupture-free balloon (RFB) catheter, made of chloroethylene, to prevent rupture and increase visibility by ultrasonography. In principle, we intravenously administered midazolam and pentazocine for conscious sedation and tranquilization.

**Fig. 1:**
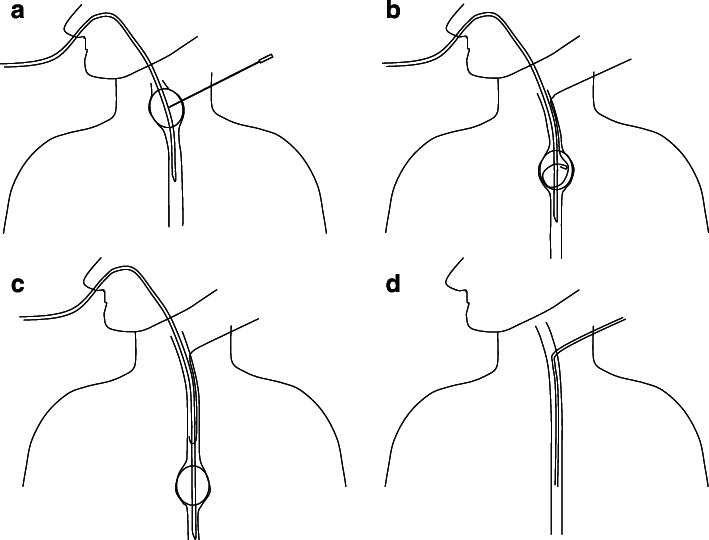
**a** The RFB catheter was inserted via the nasal cavity. It was inflated and pulled up; we punctured the RFB at the cervical area, through the esophagus. **b** The wire was inserted into the RFB through the needle. **c** RFB was pushed toward the caudal side, and the guidewire was released. **d** The tube was inserted along the wire

The RFB catheter was inserted via the nasal cavity, with the patient supine on the fluoroscopy table. It was inflated with dilute contrast material and pulled up; we punctured the RFB at the cervical area, through the cervical esophagus, under ultrasonographic vision (Fig. 1a). The wire was inserted into the RFB through the needle (Fig. 1b) and pushed toward the caudal side. After the guide wire was released from the RFB (Fig. 1c), we removed only the RFB, leaving the wire in the esophagus. The dilator included in the kit is inserted over the guide wire and the track was dilated. Next, the guide wire was removed, and the sheath remained. Subsequently, the tube was inserted depending on each application, and fluoroscopy confirmed the end of the procedure (Fig. 1d).

### Indication

PTEG is positioned as an alternative for PEG; it is indicated for the patients for which PEG is not indicated, i.e., after gastrectomy, with massive ascites, or severe gastroesophageal reflux. Contraindications for PTEG are esophageal and cervical organ disorders [1].

### Case 1

**Fig. 2:**
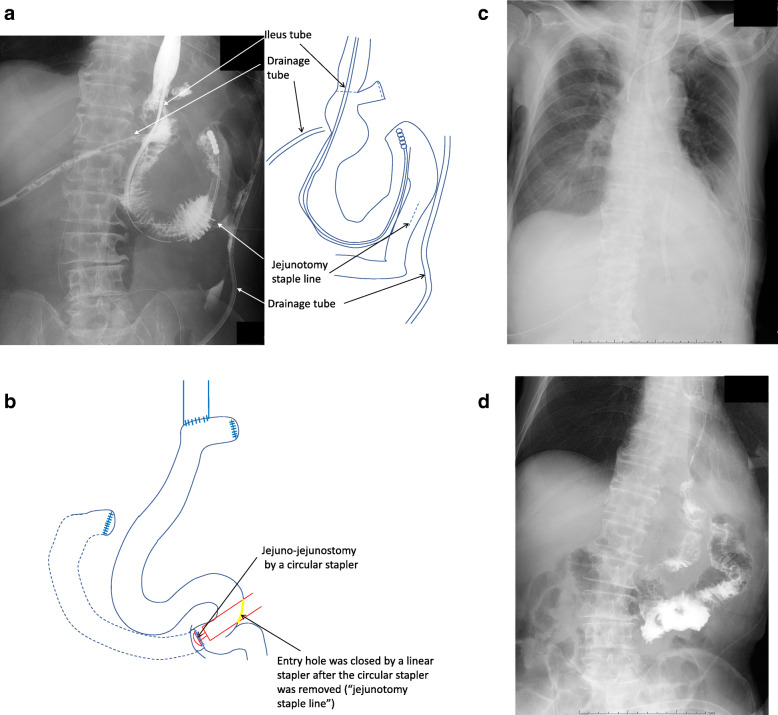
Case 1. **a** The contrast medium leaked through the drainage tubes into the esophagojejunostomy and the jejunotomy staple line (arrows). **b** The scheme shows jejunojejunal anastomosis with a circular stapler. We made an incision at the side of the elemental limb, inserted circular stapler, and anastomosed end to side; the jejunotomy staple line was made for closing the entry hole. **c** We inserted a 14-Fr catheter into the jejunal limb with side holes by esophagojejunostomy. **d** The contrast study shows that leakage stopped after effective inner drainage

A 79-year-old man with gastric cancer underwent total gastrectomy; 1 week later, he underwent emergent open laparotomy due to panperitonitis attributed to anastomotic leakage-related jejunojejunostomy. We resected the portion between the esophagojejunostomy and jejunojejunostomy, due to severe edema of the jejunal limb. We reconstructed it using the Roux-en-Y technique and put drainage tubes at the subphrenic and adjacent esophagojejunostomy areas. On postoperative day (POD) 9, he had a high fever and leukocytosis with contaminated output through the drainage tubes. We diagnosed anastomotic leakage at the esophagojejunostomy site and jejunotomy staple line (Fig. 2a). At the emergency operation, we tried to make Roux-en-Y reconstruction again. After the necrotic intestine was removed, the duodenal limb was not long enough for anastomosis by linear stapling or hand-sewing. Therefore, we made an incision at the side of the elemental limb, inserted a circular stapler, and anastomosed end to side; the jejunotomy staple line was made for closing the entry hole (Fig. 2b). We inserted an NGT and performed low-pressure aspiration for bowel decompression. He did not improve within 2 weeks, so we decided to perform PTEG to release him from the NGT. As there were two points of anastomotic leakage, we inserted a 14-Fr catheter into the jejunal limb with side holes along the esophagojejunostomy (Fig. 2c). We kept performing intermittent aspiration; leakage stopped shortly after, due to effective inner drainage (Fig. 2d). Enteral nutrition was continued from the jejunal feeding tube inserted during surgery. Oral intake was restarted on POD 66, and the PTEG catheter was removed on POD 72.

### Case 2

**Fig. 3:**
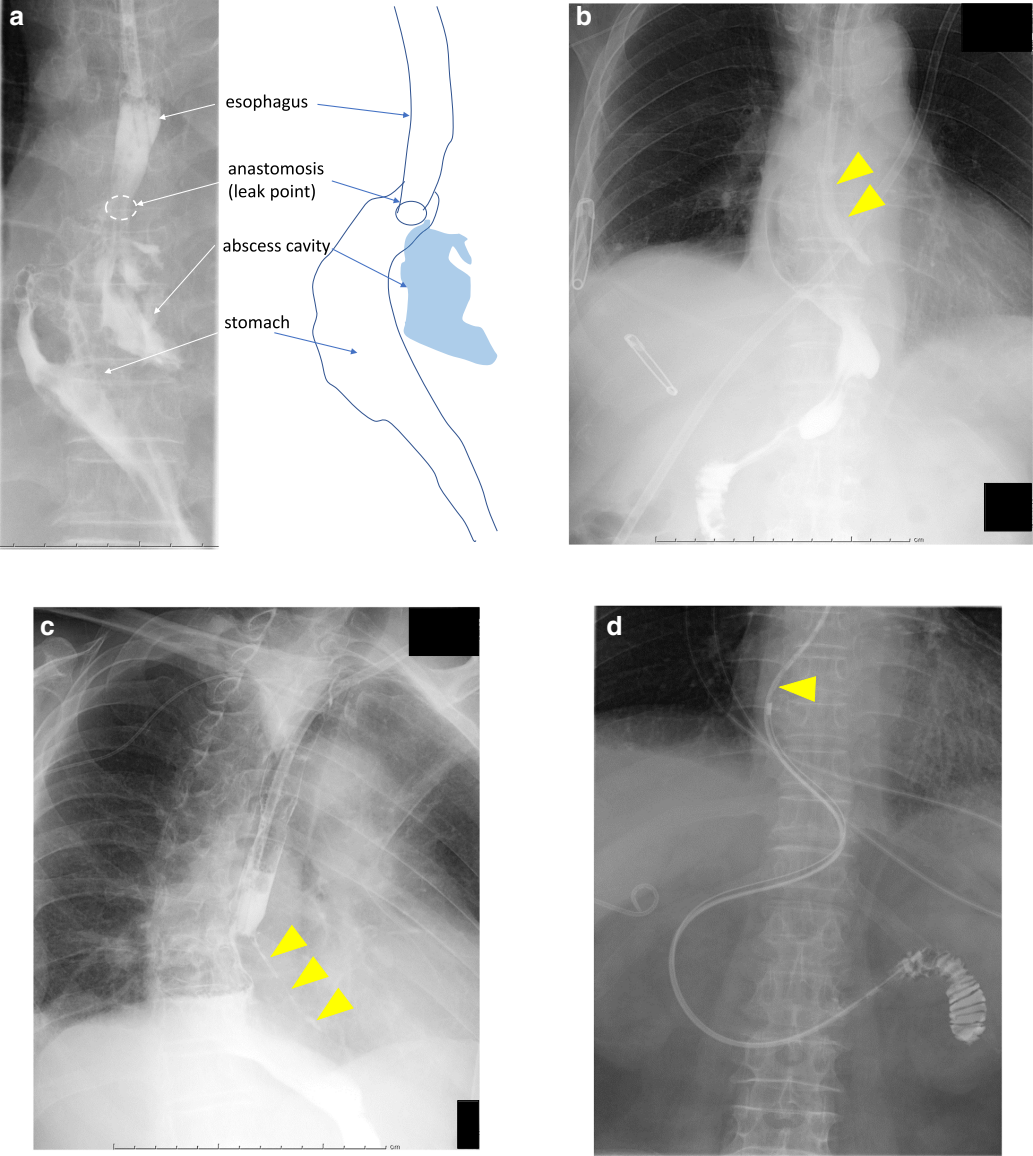
Case 2. **a** Contrast medium injection through the NGT shows the anastomotic leakage which caused the abscess. **b** We inserted the NGT into the abscess cavity (arrowheads) through the site of anastomotic leakage. **c** Even after the abscess cavity disappeared, the anastomotic leakage persisted as a fistula (arrowheads). **d** We exchanged the PTEG with a W-ED tube with jejunal extension with the side hole located near the anastomosis

An 81-year-old man with Siewert type I esophagogastric junction cancer underwent resection of the distal esophagus and proximal stomach. After shaping the remnant stomach, esophagogastrostomy was performed in the mediastinum under right thoracotomy. The anastomosis was performed using a circular stapler and was wrapped around the stomach to prevent gastroesophageal reflux disease. On POD 11, he had high fever; a computed tomography scan revealed a mediastinal abscess. The mediastinal drain was already removed on POD 9. A contrast medium injected through the NGT showed the anastomotic leakage, which caused the abscess (Fig. 3a). We inserted the NGT into the abscess cavity through the anastomotic leakage site (Fig. 3b) and ensured that the inside of the drainage tube was under negative pressure for withdrawing abscess contents by using a low-pressure aspirator. Since the tube caused pain, the patient repeatedly tried to remove it by himself. We had to restrain his limbs, so that the main drainage tube would not be removed. On POD 25, we performed PTEG and inserted a drainage tube instead of an NGT. He was soon released, after PTEG. Even after the abscess cavity disappeared, we confirmed that the anastomotic leakage persisted as a fistula, by injecting a contrast medium under fluoroscopic guidance on POD32 (Fig. 3c). As enteral feeding was required to start as soon as possible, we exchanged the PTEG tube with a double elementary diet (W-ED) tube with jejunal extension with the side hole located near the anastomosis, on POD43 (Fig. 3d). We used the 16-Fr W-ED tube, supplied by Japan Covidien Corp., Tokyo, Japan. It measures 150 cm and includes connectors both for drainage and nutrition; one lumen has its openings alongside the tube, 60 cm above the leading edge. The other lumen has its openings at the end of the tube for feeding. On POD 50, the anastomotic fistula disappeared, as seen in the fluoroscopic examination. The patient’s dysphagia persisted, due to disuse atrophy of the swallowing musculature; PTEG was useful for enteral feeding, even after the leakage occurred.

## Discussion

Anastomotic leakage after upper GI surgery sometimes causes critical conditions, if left without prompt and appropriate treatment. If the drainage tube is inserted during surgery, it can certainly be used. However, unfortunately, if anastomotic leakages occur after the drainage tubes are removed or are not effective for removing leaked intestinal juice or pus, another drainage pathway needs to be added. Kosumi et al. have reported about transnasal inner drainage for the management of post-esophagectomy leakage [23]. Thus, a drainage tube can be inserted through the site of anastomosis without surgical treatment or percutaneous puncture, guided by ultrasonography or fluoroscopy. Even though it is difficult to reach the tube into the abscess cavity, intraesophageal pressure can be relieved by continuous suction. Additionally, transnasal inner drainage is effective in terms of minimal invasion, affordability, and technical simplicity.

However, a long period is required until the tube is removed. Long-term use of an NGT causes poor patient tolerability and complications, including epistaxis, aspiration pneumonia, and sinusitis. Oshiro et al. have reported PTEG for the management of gastric leakage after sleeve gastrectomy [24]. For patients who underwent bariatric surgery, it is challenging to re-operate for anastomotic leakage because of the patients’ risk, i.e., they are highly obese and therefore have many preoperative complications. Though bowel decompression using a nasal tube is less invasive than re-operation, nasal discomfort is a severe problem for patients. Repeated surgical treatments were also difficult to perform in this report because the patients were emaciated. In case 1, the patient was originally bedridden and malnourished. In case 2, the patient was elderly, had severe diabetes, and was on steroids. In terms of inner drainage and bowel decompression, PTEG is useful not only for sleeve gastrectomy, but every type of upper GI surgery.

Oshiro et al. also described that endoscopic stenting is a well-established and viable option for enabling the healing of a fistula opening. A benefit of endoluminal stenting is that patients may be able to resume oral intake and be discharged after stenting. However, insufficient success and premature stent removal because of insufficient sealing, migration, and obstruction have been reported [25, 26].

In addition to drainage, the purpose of PTEG is to maintain a route of access for enteral feeding. Patients with anastomotic leakages cannot receive long-term enteral feeding without distal feeding access. As in our second case, the patient had to endure a fasting period. Using a double-lumen tube, we could initiate simultaneous enteral feeding and bowel decompression around the anastomosis. Iwase et al. have proposed double PTEG, namely 2 PTEG tubes placed through the same orifice, simultaneously [27]. They have safely performed double PTEG, and we thought it would be particularly effective for our patients. However, placing two tubes simultaneously is a rather complex procedure. As we have found that a W-ED tube is used for a patient with spontaneous esophageal perforation [28], we thought it could apply to PTEG. While the inner diameter is smaller than double PTEG tubes, it is certainly easier to insert, as the size of the W-ED tube just fits the cervical fistula.

The ESPEN guideline describes that malnutrition and undernutrition are risk factors for postoperative complications, and early enteral feeding is especially relevant for any surgical patient at nutritional risk, especially for those undergoing upper gastrointestinal surgery [29]; PTEG can offer the benefit of early commencement of feeding without an NGT.

In this way, we have added a wide variety of applications to PTEG. Two successful cases we experienced suggest PTEG has many possibilities. Although the PTEG procedure is unique, it is not complex to perform, once its methodology is known; PTEG was performed on many patients for the decompression of malignant bowel obstruction, or as a feeding access site for those who are unable to receive percutaneous endoscopic gastrostomy (PEG). Certainly, some complications should be expected; however, it is a relatively safe and feasible procedure when being familiar with the anatomy of the cervical area and exercise caution, as with other surgical treatments.

## Conclusion

We successfully managed anastomotic leakage after upper GI surgery using PTEG. We believe that PTEG should be helpful for almost all patients withstanding the NGT for a long time.

## Data Availability

Not applicable.

## Abbreviations

GI: Gastrointestinal
NGT: Nasogastric tube
PEG: Percutaneous endoscopic gastrostomy
PTEG: Percutaneous transesophageal gastro-tubing
RFB: Rupture-free balloon
